# The association between *Helicobacter pylori* seropositivity and acne vulgaris among adolescent schoolchildren in Sudan: a case-control study

**DOI:** 10.25122/jml-2026-0110

**Published:** 2026-07

**Authors:** Moteb Alotaibi, Ishag Adam

**Affiliations:** 1 Department of Dermatology, College of Medicine, Qassim University, Buraydah, Saudi Arabia; 2 Department of Obstetrics and Gynecology, College of Medicine, University of Khartoum, Khartoum, Sudan

**Keywords:** acne vulgaris, *Helicobacter pylori*, adolescents, seroprevalence, case-control study, Sudan

## Abstract

Acne vulgaris affects an estimated 9.4% of the world's population and is the eighth most prevalent disease globally. *Helicobacter pylori* has been implicated in several inflammatory dermatoses, but the evidence linking it to acne rests on a small number of clinic-based studies, and no data have been reported from Africa. We examined this association in a school-based case-control study of adolescents aged 10–19 years attending public schools in Almatamah Locality, northern Sudan, between October and November 2022. Acne was diagnosed and graded by trained physicians using the Global Acne Grading System, and *H. pylori* IgG/IgM antibodies were detected by a rapid immunochromatographic assay. Groups were compared using the χ^2^, Mann–Whitney, and Fisher–Freeman–Halton exact tests, and associations were estimated by univariate and multivariable logistic regression. Of 313 adolescents enrolled, 130 had acne (99 [76.2%] mild, 28 [21.5%] moderate, and 3 [2.3%] severe) and 183 did not. Seroprevalence was higher among adolescents with acne (19/130, 14.6%; 95% CI, 9.6–21.7) than among those without (5/183, 2.7%; 95% CI, 1.2–6.2), a difference of 11.9 percentage points (95% CI, 5.7–19.1; *P* < 0.001). Female sex (adjusted odds ratio [AOR] 2.80; 95% CI, 1.72–4.56) and *H. pylori* seropositivity (AOR 5.28; 95% CI, 1.86–14.94) were independently associated with acne, whereas body mass index was not. Among adolescents with acne, seropositivity was not associated with severity (*P* = 0.850). *H. pylori* seropositivity was associated with acne vulgaris in this population, but the absence of a severity gradient and the small number of exposed participants warrant cautious interpretation.

## Introduction

Acne vulgaris is a chronic inflammatory disorder of the pilosebaceous unit. It is estimated to affect 9.4% of the global population, which makes it the eighth most prevalent disease worldwide [1,2]. More than 85% of adolescents are affected at some point, and in a substantial minority the disease persists into adulthood [3]. Reported prevalence among adolescents in African countries varies widely, from 28% in Mali to 59% in Cameroon, with intermediate figures of 44.4% in Ghana and 53.2% in Nigeria [4]. Although acne is not life-threatening, it carries a measurable psychosocial burden affecting self-esteem, social functioning, and mental health [5].

Acne is multifactorial: genetic susceptibility, pubertal androgen exposure, diet, environmental factors, stress, and medications all contribute [3]. Its pathogenesis involves increased sebum production, follicular hyperkeratinization, colonization and proliferation of *Cutibacterium acnes*, and a consequent inflammatory response [3,6]. Lesions are conventionally divided into non-inflammatory (open and closed comedones) and inflammatory (papules, pustules, nodules, and cysts) types [3,6].

*Helicobacter pylori* is a gram-negative, flagellated bacterium that colonizes the gastric epithelium and is causally linked to peptic ulcer disease, gastric adenocarcinoma, and gastric mucosa-associated lymphoid tissue lymphoma [7]. Its global prevalence has been estimated at 43.1%, with the highest regional estimates reported for Africa [8]; antimicrobial resistance and limited diagnostic access complicate its management across the continent [9]. *H. pylori* has also been associated with a range of extragastric conditions, including several inflammatory dermatoses such as Behçet disease, psoriasis, chronic urticaria, and rosacea [10].

Evidence linking *H. pylori* specifically to acne is far less conclusive. A small number of clinic-based studies have reported higher rates of *H. pylori* infection in patients with acne, and in some cases a gradient with acne severity [7,11–13]. No study to date has been conducted in Africa, and none has used a community-based adolescent sample. Because acne and *H. pylori* infection are both common in this age group and share plausible sociodemographic determinants, community data are needed before any inference about a causal or therapeutic relationship can reasonably be drawn. We therefore examined the hypothesis that *H. pylori* seropositivity is associated with acne vulgaris among adolescents attending public schools in northern Sudan.

## Material and Methods

### Study design and setting

This was an observational, school-based case-control study conducted between October and November 2022 in Almatamah Locality, approximately 130 km from Khartoum, Sudan. The study was local in scope and involved several public schools within a single locality. We obtained a comprehensive list of public schools and enrolled students aged 10–19 years from the local education authority. Schools were selected at random from this list, and eligible students were then selected at random within the selected schools. Clinical examination and blood sampling were carried out by trained physicians.

The present analysis draws on the same field survey, participants, and ethical approval as a previously published report describing the prevalence and correlates of *H. pylori* infection in this adolescent population [14]. That report addressed *H. pylori* infection as the outcome and did not assess acne vulgaris, which was neither an exposure nor an outcome in that analysis. The present study addresses a distinct question, the association between *H. pylori* seropositivity and acne, and reports the dermatological assessment for the first time. No participant-level result is duplicated between the two papers.

### Inclusion and exclusion criteria

Eligible participants were students aged 10–19 years enrolled in the selected schools. Students were excluded if they were outside the specified age range, were acutely unwell, were pregnant or breastfeeding, or if they or their guardians declined to participate. Cases were adolescents with clinically diagnosed acne vulgaris; controls were adolescents from the same schools with no clinical evidence of acne.

### Assessment of acne vulgaris

Acne vulgaris was diagnosed clinically and graded by the Global Acne Grading System (GAGS) [15]. The GAGS divides the body into six regions: forehead, right cheek, left cheek, nose, chin, and chest with upper back. Within each region, the most severe lesion type present is scored (no lesio*n* = 0, comedone = 1, papule = 2, pustule = 3, nodule = 4) and multiplied by a region-specific factor (×2 for forehead and each cheek; ×1 for nose and chin; ×3 for chest and upper back). Regional scores are summed to give a global score, categorized as none (0), mild (1–18), moderate (19–30), severe (31–38), or very severe (>38) [15]. Participants with a global score of 0 were classified as free of acne. Physicians performing the dermatological assessment were blinded to *H. pylori* results, which were generated after clinical examination.

### *H. pylori* serology

Venous blood (3 mL) was collected from each participant. Samples were centrifuged at 1500 rpm for 15 minutes, and serum was tested with a rapid immunochromatographic *H. pylori* antibody test (Hangzhou AllTest Biotech Co., Ltd., Hangzhou, China) according to the manufacturer’s instructions. The assay detects *H. pylori*-specific IgG and IgM antibodies; the manufacturer reports a sensitivity of 95.5% and a specificity of 91.3% [16]. Results were recorded as positive or negative.

### Anthropometric and sociodemographic data

Weight was measured to the nearest 100 g using calibrated scales zeroed before each measurement, and height to the nearest 1 mm. Body mass index (BMI) was calculated as weight in kilograms divided by height in meters squared. A structured questionnaire recorded age in years, sex, maternal and paternal educational attainment (secondary or above versus below secondary), maternal employment (housewife versus employed), and paternal occupation (skilled versus non-skilled).

### Endpoints and variables

The primary endpoint was the presence of acne vulgaris (present versus absent). The secondary endpoint was acne severity category (mild, moderate, severe) among participants with acne. The exposure of interest was *H. pylori* seropositivity. Covariates were age (years, continuous), sex, BMI (kg/m^2^, continuous), maternal and paternal education, maternal employment, and paternal occupation.

### Sample size

Sample size was calculated using OpenEpi version 3.01 [17]. The calculation was based on a case-to-control ratio of 1:1.4, a two-sided alpha of 0.05, and 80% power. We assumed an *H. pylori* infection rate of 15.0% among cases and 7.0% among controls, based on the recently reported frequency of *H. pylori* infection among adolescents in Sudan [14]. The minimum required sample size was 222 cases and 310 controls. The achieved sample of 130 cases and 183 controls therefore provided approximately 62% power to detect the originally hypothesized effect size; however, because the observed effect size was substantially larger than assumed (Results), the achieved sample provided approximately 96% power to detect it [18].

### Statistical analysis

The data were analyzed using IBM Statistical Package for the Social Sciences for Windows, version 22.0 (IBM Corp., Armonk, NY, USA). Frequencies (percentages) were used to express proportions, and medians and interquartile ranges (IQRs) were used to express continuous data (age and BMI), which were not normally distributed. We used the Shapiro–Wilk test to assess whether the continuous data were normally distributed. χ^2^ and Mann–Whitney tests were used to compare proportions and medians, respectively, between adolescents with acne and those without acne; for the comparison of acne severity by *H. pylori* status among adolescents with acne, the Fisher–Freeman–Halton exact test was used in place of the χ^2^ test because several expected cell counts were below 5. In the univariate analysis, acne vulgaris was the dependent variable, and the independent variables were age, sex, parental education and occupation, BMI, and *H. pylori* serostatus. To account for confounders, variables with a *P* value below 0.20 in the univariate analysis were entered into a multivariable logistic regression model (backward conditional elimination). Crude and adjusted odds ratios (CORs and AORs) with 95% confidence intervals were computed. We calculated the E-value to quantify the strength of unmeasured confounding required to explain away the adjusted association. A two-sided *P* value below 0.05 was considered statistically significant.

## Results

A total of 313 adolescents were enrolled: 130 with acne vulgaris and 183 controls. Among adolescents with acne, 99 (76.2%) had mild, 28 (21.5%) moderate, and 3 (2.3%) severe disease; no participant met the criteria for very severe acne. Cases and controls did not differ significantly in age, parental education, or parental occupation (Table 1). The proportion of girls was higher among cases than among controls (90/130 [69.2%] versus 77/183 [42.1%]; *P* < 0.001), as was the median (IQR) body mass index (19.0 [17.5–21.7] versus 18.4 [16.4–21.3] kg/m^2^; *P* = 0.033).

**Table 1:** Characteristics of adolescent schoolchildren in northern Sudan, 2022, by acne status, and crude associations with acne vulgaris (*n* = 313)

Variable	Category	Acne (*n* = 130)	No acne (*n* = 183)	Crude OR (95% CI)	*P*
Age, years	Median (IQR)	15.3 (14.4–16.0)	15.0 (13.9–16.0)	1.09 (0.93–1.27)	0.265
Body mass index, kg/m^2^	Median (IQR)	19.0 (17.5–21.7)	18.4 (16.4–21.3)	1.07 (1.01–1.14)	**0.033**
Sex	Male	40 (30.8)	106 (57.9)	Reference	—
	Female	90 (69.2)	77 (42.1)	3.10 (1.93–4.98)	**< 0.001**
Mother’s education	Secondary or above	80 (61.5)	115 (62.8)	Reference	—
	Below secondary	50 (38.5)	68 (37.2)	1.06 (0.67–1.68)	0.815
Mother’s occupation	Housewife	116 (89.2)	170 (92.9)	Reference	—
	Employed	14 (10.8)	13 (7.1)	1.58 (0.72–3.48)	0.258
Father’s education	Secondary or above	87 (66.9)	121 (66.1)	Reference	—
	Below secondary	43 (33.1)	62 (33.9)	0.96 (0.60–1.55)	0.882
Father’s occupation	Skilled	50 (38.5)	70 (38.3)	Reference	—
	Non-skilled	80 (61.5)	113 (61.7)	0.99 (0.62–1.57)	0.970
*H. pylori* serology	Negative	111 (85.4)	178 (97.3)	Reference	—
	Positive	19 (14.6)	5 (2.7)	6.09 (2.21–16.79)	**< 0.001**

Data are presented as *n* (%) unless otherwise stated. Comparisons were made using the Mann–Whitney test for continuous variables and the χ^2^ test for categorical variables. Odds ratios and confidence intervals were obtained by univariate logistic regression and are given per one-unit increase for continuous variables. Bold indicates *P* < 0.05. An em dash indicates not applicable. CI, confidence interval; IQR, interquartile range; OR, odds ratio.

*H. pylori* seroprevalence was 14.6% among cases (19/130; 95% CI, 9.6–21.7) and 2.7% among controls (5/183; 95% CI, 1.2–6.2), a difference of 11.9 percentage points (95% CI, 5.7–19.1; χ^2^ *P* < 0.001; Table 2). Of the 19 seropositive adolescents with acne, 14 had mild, and 5 had moderate disease; the remaining 111 were seronegative (Table 3). Age, parental education, and parental occupation were not associated with acne vulgaris in the univariate analysis. Female sex (COR 3.10; 95% CI, 1.93–4.98), a one-unit increase in body mass index (COR 1.07; 95% CI, 1.01–1.14), and *H. pylori* seropositivity (COR 6.09; 95% CI, 2.21–16.79) were associated with acne vulgaris in this analysis (Table 1).

**Table 2:** Multivariable logistic regression of factors associated with acne vulgaris among adolescent schoolchildren in northern Sudan, 2022 (*n* = 313)

Variable	Category	Adjusted OR (95% CI)	*P*
Body mass index, kg/m^2^	Per 1-unit increase	1.06 (0.99–1.14)	0.078
Sex	Male	Reference	—
	Female	2.80 (1.72–4.56)	**< 0.001**
*H. pylori* serology	Negative	Reference	—
	Positive	5.28 (1.86–14.94)	**0.002**

Variables with *P* < 0.20 in the univariate analysis were entered into a multivariable logistic regression model with backward conditional elimination. Bold indicates P < 0.05. An em dash indicates not applicable. AOR, adjusted odds ratio; CI, confidence interval; OR, odds ratio.

In the multivariable model, female sex (AOR 2.80; 95% CI, 1.72–4.56) and *H. pylori* seropositivity (AOR 5.28; 95% CI, 1.86–14.94) remained independently associated with acne vulgaris, whereas body mass index did not (AOR 1.06; 95% CI, 0.99–1.14) (Table 2). The E-value for the adjusted odds ratio was 4.02, and 2.07 for its lower confidence limit: an unmeasured confounder would need to be associated with both *H. pylori* seropositivity and acne by a risk ratio of at least 4.02 each, above and beyond the measured covariates, to nullify the point estimate.

Among the 130 adolescents with acne, *H. pylori* seropositivity was not associated with acne severity (Fisher–Freeman–Halton exact *P* = 0.850). All three participants with severe acne were seronegative (Table 3).

**Table 3:** Acne severity by Helicobacter pylori serostatus among 130 adolescents with acne vulgaris

Acne severity (GAGS score)	*H. pylori* positive (*n* = 19)	*H. pylori* negative (*n* = 111)	Total (*n* = 130)
Mild (1–18)	14	85 (76.6)	99 (76.2)
Moderate (19–30)	5	23 (20.7)	28 (21.5)
Severe (31–38)	0	3 (2.7)	3 (2.3)

Data are presented as *n* (%). Serostatus groups were compared using the Fisher–Freeman–Halton exact test (*P* = 0.850). No participant had very severe acne (GAGS score > 38). GAGS, Global Acne Grading System.

## Discussion

In this community-based sample of Sudanese adolescents, *H. pylori* seropositivity was associated with acne vulgaris, with an adjusted odds ratio of 5.28. To our knowledge, these are the first data on this question from Africa and the first from a non-clinical adolescent population. Three features of the result nevertheless call for caution before interpreting it as evidence of a causal relationship.

First, the association shows no gradient with disease severity. Among adolescents with acne, seropositivity was no more common in moderate than in mild disease, and all three participants with severe acne were seronegative (*P* = 0.85). This matters because a dose-response relationship is among the strongest observational arguments for causality, and its absence here contrasts with the clinic-based studies on which the hypothesis largely rests. Saleh *et al*. reported higher *H. pylori* antigen and antibody levels in severe than in mild and moderate acne [11]; Khodaeiani *et al*. reported infection rates rising from 60% in mild acne to 72% in moderate and 88% in severe acne [12]; and Afify *et al*. likewise found rates increasing with severity [13]. The most likely explanation for the discrepancy is limited power, as only 19 seropositive cases and three severe cases were available; but an absent gradient in a community sample where earlier clinic samples found one is a finding worth stating plainly rather than setting aside.

Second, the observed seroprevalence is difficult to reconcile with regional estimates. At 7.7% overall and 2.7% among adolescents without acne, our figures are an order of magnitude below pooled African estimates for *H. pylori* [8], although they are closely consistent with the 8.4% we previously reported using the same assay in this population [14]. Two explanations deserve consideration. The assay may have limited sensitivity in this setting, in which case a substantial proportion of adolescents classified as seronegative were in fact infected. If such misclassification were non-differential, it would bias the odds ratio towards the null, and the true association could be larger than reported. If, however, test strips were read with knowledge of acne status, misclassification could be differential and the association spuriously inflated. Alternatively, antibody titers in early adolescence may not yet have reached detection thresholds, or local seroprevalence may genuinely be lower than regional pooled figures suggest. Distinguishing these possibilities would require a validated reference standard such as the urea breath test or a stool antigen assay.

Third, the published literature relating *H. pylori* to acne consists of a small number of studies, nearly all reporting positive associations. In a field this small, the absence of null reports is more likely to reflect publication practice than a genuine absence of null findings, and readers should weigh the existing evidence base accordingly.

Female sex was independently associated with acne in this sample (AOR 2.80). This requires careful handling, because sex is a common cause of both acne and *H. pylori* infection [14]. It should also be noted that the direction of the sex association in adolescence is not uniform in the literature. Age-standardized global estimates show a higher burden in females [19], and studies in Egyptian and Saudi adolescents report a female predominance [20,21]; however, Tan and Bhate report that acne in postpubescent teenagers is most frequent in boys, particularly in more severe forms [2]. Differential participation may also contribute, in that girls may be more willing than boys to be examined for a visible skin condition.

If the association between *H. pylori* and acne is real, several mechanisms are plausible. *H. pylori* infection generates a systemic inflammatory response involving nitric oxide and other mediators [22], and induces interleukin-8, tumor necrosis factor-alpha [23], and calprotectin [24,25], the last of which has been reported at elevated levels in acne. A more speculative connection comes from Ryu *et al*., who reported that HPA3NT3, a synthetic antimicrobial peptide derived from *H. pylori*, has antibacterial and anti-inflammatory activity against *C. acnes* [26], though this points, if anything, in the opposite direction. Khashaba *et al*. reported reductions in lesion counts following eradication therapy in a prospective cohort [7], which would be the most direct evidence of a causal link, but that study was uncontrolled, and lesion counts in acne fall substantially with time and attention alone. None of these mechanisms has been tested in a design capable of establishing causality, and we regard the pathway as unresolved.

### Strengths and limitations

The main strengths of this study are its community-based design, the random selection of schools and students within schools, the use of a standardized grading instrument applied by trained physicians blinded to serological results, and the reporting of an E-value to quantify sensitivity to unmeasured confounding. These features reduce the referral and detection biases that affect clinic-based samples.

Several limitations should be considered. The case-control design cannot establish that *H. pylori* infection preceded the development of acne, and the possibility of reverse causation, though unlikely, cannot be ruled out. Serological testing identifies exposure rather than current infection and cannot distinguish active from cleared infection; the low observed seroprevalence suggests the test’s sensitivity may be insufficient for this population. The association estimate is based on a limited number of seropositive participants, resulting in low precision. Pubertal stage, dietary intake, and household-level variables were not measured and may confound the association. As the study was confined to a single locality, the findings may not be generalizable to other regions of Sudan. Finally, recruitment did not reach the intended sample size.

## Conclusion

*H. pylori* seropositivity was associated with acne vulgaris among adolescent schoolchildren in northern Sudan. Although the association was strong, it is based on a small number of exposed participants, shows no gradient with disease severity, and derives from a design that cannot establish temporality. These findings are therefore suggestive rather than conclusive. They warrant further investigation in longitudinal studies using a standardized diagnostic test, with measurement of pubertal stage and household variables, and they do not, in themselves, justify testing for or eradicating *H. pylori* as part of acne management.

## Data Availability

Further data are available from the corresponding author upon reasonable request.
